# A History of Endoscopic Lumbar Spine Surgery: What Have We Learnt?

**DOI:** 10.1155/2019/4583943

**Published:** 2019-04-03

**Authors:** H. Michael Mayer

**Affiliations:** ^1^Spine Center, Schön Klinik München Harlaching, Academic Teaching Hospital, Harlachinger Str. 51, 81547 München, Germany; ^2^Institute for Spinal Research, Paracelsus Medical University, Salzburg, Austria

## Abstract

The new development and finally the general acceptance of surgical techniques among the worldwide surgical community sometimes create fascinating stories. This is also true for the history of endoscopic lumbar spine surgery. In the last 100 years there was a “natural” evolution of surgical techniques with continuous improvement and “refinement” of lumbar decompression techniques towards less invasive operations with the final “endpoint” of microsurgery. However the application of percutaneous, image-guided, and endoscopic technologies has revolutionized minimally invasive surgery. This article describes the history of endoscopic lumbar spine surgery and its major milestones and protagonists which have helped to make endoscopic lumbar spine surgery “disruptive” minimally invasive surgical technology which has changed the world of lumbar decompression surgery.

“The past is the mother of the future”

Henri Cartier Bresson, French Photographer, 1908-2004

## 1. Introduction

Development and progress in spinal surgery have always been characterized by “back-and-forth movements” in clinical applications of technical innovations. Most evolutionary technical improvements which seemed to have a logical indication spectrum, with adequate feasibility and a perspective to improve early or late outcomes, have sooner or later become “standard” with a worldwide market penetration. A good example of such a development is anterior cervical discectomy and fusion (ACDF). It all started with the Cloward and Smith-Robinson technique [1, 2], which was improved with the development of plates [3–5] to support and fix the bone grafts. The bone grafts were replaced by cages made from different materials, and further technical improvement has led to the use of cages as stand-alone devices recently. This is a typical simple example of a continuous evolution of a surgical technique.

The lesson we can learn from this is that if a technical improvement follows the needs of the surgeon and if it improves or standardizes a surgical technique and its outcomes, the acceptance among the surgical community will be logical and high.

## 2. History of Lumbar Disc Surgery

### 2.1. Part 1: From Complete Laminectomy to Microsurgical/Microendoscopic Techniques

The history of lumbar discectomy and lumbar decompression is one of the most fascinating chapters of spine surgery which has taught us a number of important lessons.

**Figure 1:**
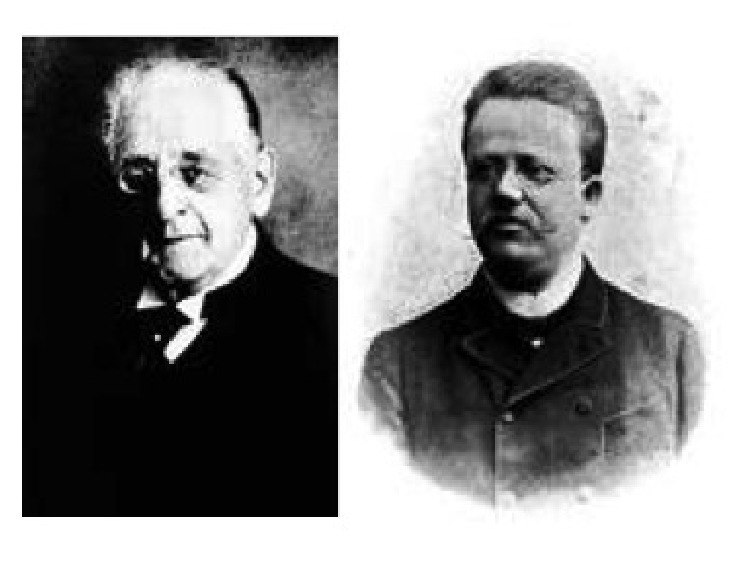
F Krause and H Oppenheim: first surgical removal of a “chondroma” of the spinal canal 1909.

**Figure 2:**
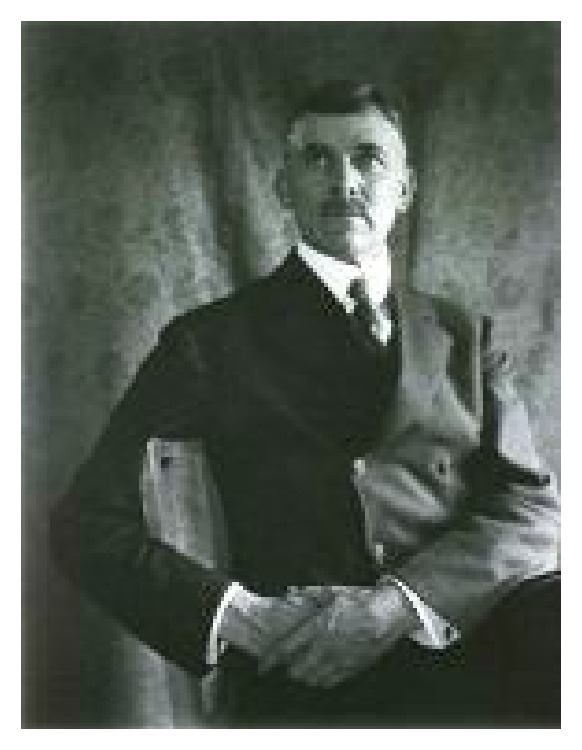
JE Goldthwaite: first description of herniated nucleus pulposus as reason for sciatica, 1911.

It was in 1909 when Krause and Oppenheim described the first lumbar discectomy [6] (Figure 1). Erroneously they described the herniated disc as a chondroma of the lumbar spinal canal. Only 2 years later Goldthwaite and Middleton were the first to describe a herniated nucleus pulposus as a reason of low back pain and sciatica [7, 8](Figure 2)

**Figure 3:**
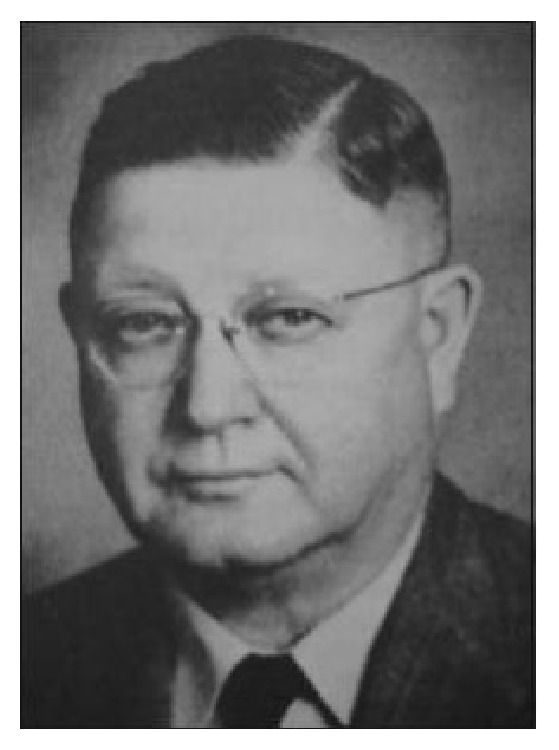
AW Adson: first description of surgical removal of herniated nucleus pulposus, 1922.

And it took another 11 years until Adson came up with the first report about surgical removal of herniated nucleus pulposus [9](Figure 3).

**Figure 4:**
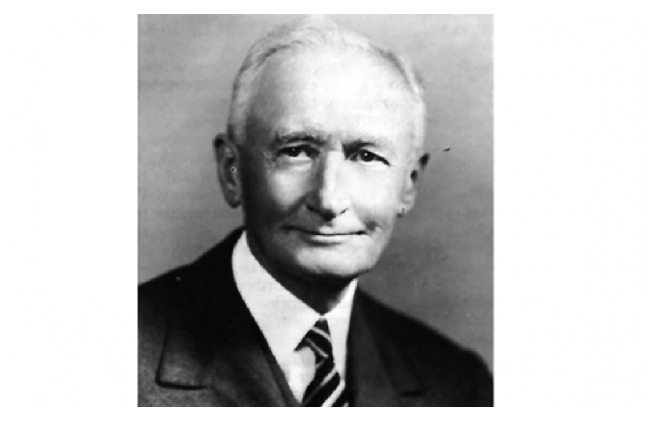
WJ Mixter: first case series of surgical removal of herniated discs 1934.

However, like very often in medical history the merits for the first disc surgeries went to two other colleagues, namely, Mixter and Barr, who still are considered as having been the “first disc surgeons” in 1934 [10] (Figure 4). They actually published the first series of successful disc operations in 1934. Their technique however was a complete laminectomy and some of the disc herniations were removed through a transdural approach.

It was obvious from the beginning that this was a very traumatic approach with the potential of a variety of complications including dural leaks and segmental instability as well as disabling back pain.

**Figure 5:**
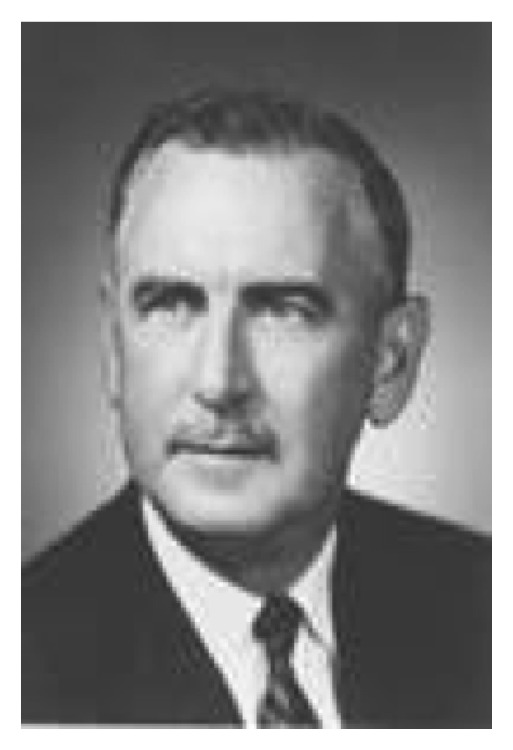
JG Love: first description of interlaminar approach, 1939.

The search for less damaging approaches had started. Only 5 years later, Love described the first interlaminar approach [11] which became the standard procedure for many years (Figure 5). But even though the rate of major surgical complications dropped over time, the problem of postoperative back pain and rapid progression of disc degeneration due to aggressive disc removal affected the clinical outcomes.

While surgery led to a significant improvement of nerve root compression signs, patient satisfaction was impaired by symptoms which were due to the collateral damage the surgeon had produced. Interestingly this fear is still immanent in today's public opinion about disc surgery.

**Figure 6:**
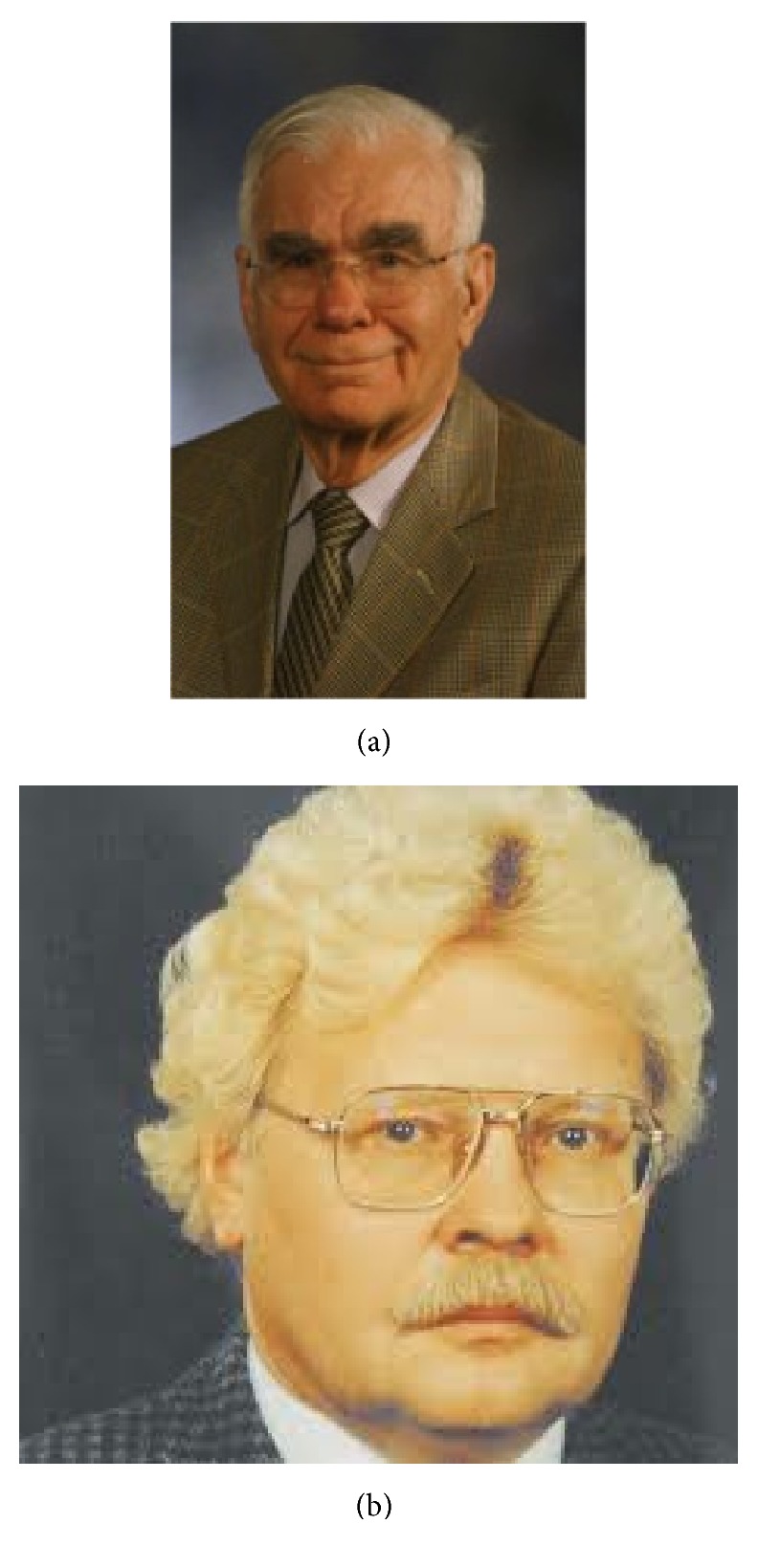
(a) G Yasargil, (b) W Caspar: first description of microsurgical interlaminar approach.

The reduction of collateral damage was the driving force for the two pioneers of lumbar microsurgery. In the same year 1977 Yasargil and Caspar described independently a microsurgical interlaminar approach [12, 13], Figures 6(a) and 6(b). One year later, it was “Tex” Williams who was the first surgeon to perform this approach in the US [14]. The pioneering work of JA McCulloch made this approach popular in the 90s of the last century and it has become a “gold standard” at least in the neurosurgical community worldwide [15]. Other approaches such as the lateral extraforaminal access have been described in this book as well.

“Microendoscopic discectomy” was described in the beginning of this century as a modification of the microsurgical technique where the surgical microscope is replaced by “open” endoscopy [16]. This technique however did not add any further technical or clinical advantages. However both minimally invasive techniques are practiced with good and reproducible clinical outcomes [17].

### 2.2. Lessons Learnt from Microsurgical Techniques

In summary lumbar microsurgery has significantly improved clinical short-term outcomes of lumbar discectomy mainly by reducing iatrogenic collateral damage. Thus, hospitalization times have become shorter, postop pain levels are lower, and intraoperative blood loss as well as the risk of infection is less.

Even though the advantages are obvious, several lessons had to be learnt by the protagonists of such techniques.

Since there is obviously no effect on the long-term outcome of lumbar discectomy, the acceptance especially by the older generation of spine surgeons has been low despite the obvious advantages.

It has been known for many years that long-term outcome of lumbar discectomy has different predictors than the short-term outcome [18]. This is due to the fact that there is a progressive degeneration of the spine which can cause clinical symptoms at other levels which are not related to a previous disc surgery.

However we have learnt that one of the strongest predictors of a good long-term outcome is a good short-term outcome. And we have also learnt that a good short-term outcome is predicted by 2 factors: (1) the efficacy of nerve root compression and (2) the extent of iatrogenic collateral damage to muscles, ligaments, facet joints, nerve, and epidural space.

### 2.3. Part 2: The “Parallel World” of “Percutaneous” and Endoscopic Techniques

It was in 1964 when Lyman Smith published a paper about enzymatic dissolution of the nucleus pulposus, a procedure which he called chemonucleolysis [19]. It was known at that time that an enzyme called Chymopapain, which was derived from the papaya plant, was able to hydrolyze proteoglycans. During experimental work in the 50s of the last century about the effects of papain, there was an interesting incidental finding. Intravenous injection of papain in rabbits resulted in a reversible collapse of rabbit ears [20], a finding which suggested an effect of this enzyme on cartilage. Similar effects were then reported on cartilage of joints, trachea, larynx, and bronchi. Since further studies on rabbits had shown that this enzyme dissolves the nucleus pulposus [21], it was Lyman Smith's idea that an application in contained disc herniations could lead to an “intradiscal decompression”, thus relieving the symptoms from nerve compression due to a bulging lumbar disc.

In the 1980s this procedure became popular as the least invasive technique to treat herniated lumbar discs.

Mid- to long-term outcomes were good, complications were rare, and chemonucleolysis seemed to become a viable alternative to surgical discectomy [22, 23].

**Figure 7:**
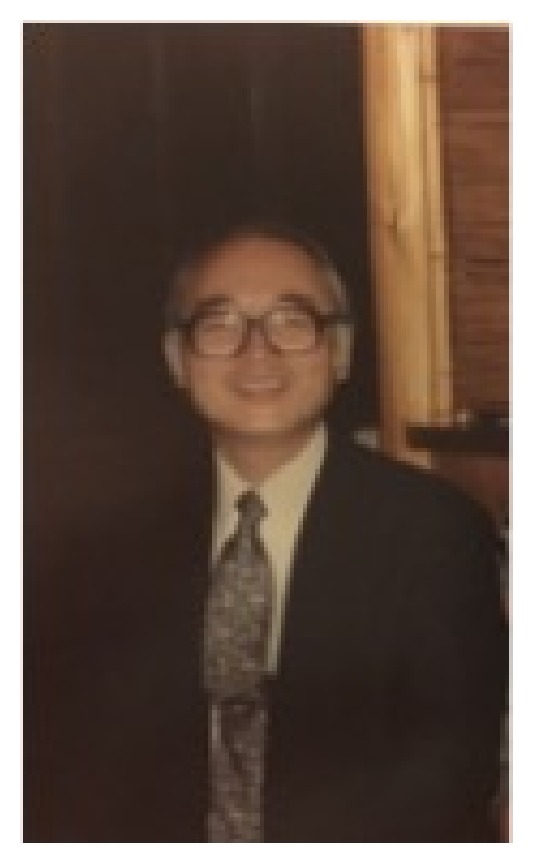
Hijikata: first percutaneous nucleotomy, 1975.

Then something happened which was more a psychological phenomenon than rational based medical evolution. In the 70s, Hijikata, a Japanese surgeon, was fascinated by the posterolateral access to the disc space which was, at that time, in the pre-CT and pre-MRI era, very popular to perform diagnostic discographies (Figure 7). He developed tubes through which he could introduce this approach down to the posterolateral annulus under fluoroscopic control. With special trephines he could perforate the annulus and, using pituitary rongeurs, he could perform what he called “percutaneous nucleotomy”. He published this procedure in a regional scientific journal in Japanese language [24]. This was one of the reasons why this procedure did not gain widespread attention among the surgical community but it was the birth of “percutaneous” and, later, endoscopic discectomy.

**Figure 8:**
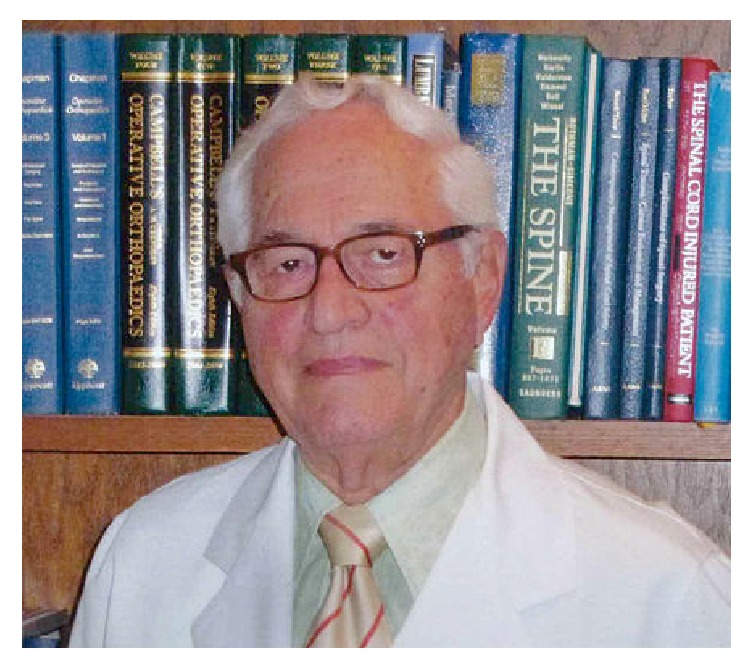
P Kambin: percutaneous discectomy, 1986.

It was the great merit of Parviz Kambin a Philadelphian spine surgeon to further develop this procedure in the 1980s [25–28] (Figure 8).

**Figure 9:**
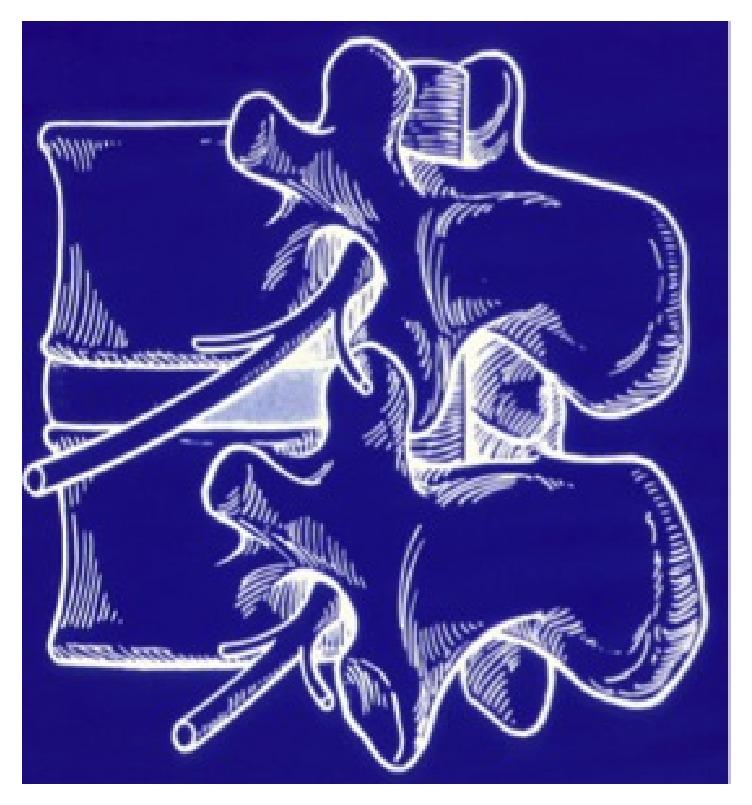
Kambin's triangle for a safe posterolateral approach.

It is the “Kambin triangle” (the safe corridor to the lumbar disc between the exiting nerve root and the superior facet) which reminds us of his pioneering work (Figure 9).

Schreiber, Suezawa, and Leu were the first to have the idea to perform this percutaneous nucleotomy under visual control using and endoscope (discoscopy) [29].

**Figure 10:**
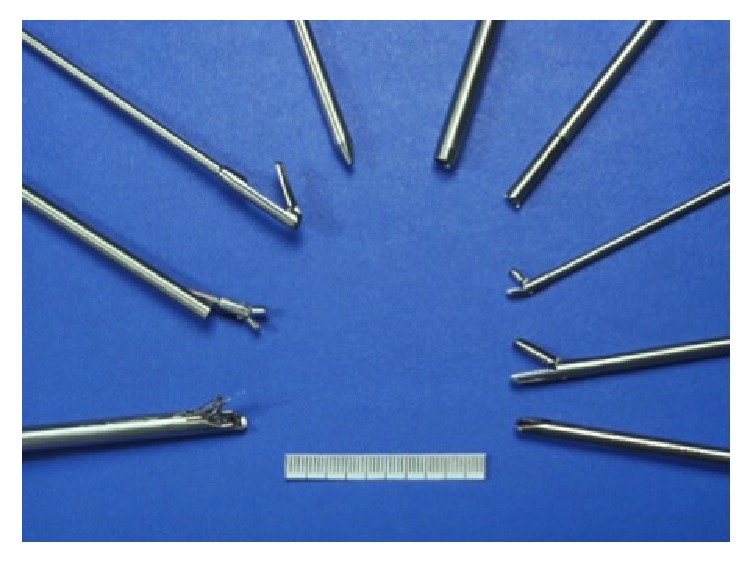
Early Instrument set for percutaneous endoscopic discectomy.

The author of this review adopted this technique, refined the instrument set [30] (Figure 10), and published the results of a randomized controlled trial comparing microdiscectomy with endoscopic posterolateral discectomy [31].

A more lateral access route was described by Hal Mathews and Tony Yeung in the second half of the 1990s [32–34].

**Figure 11:**
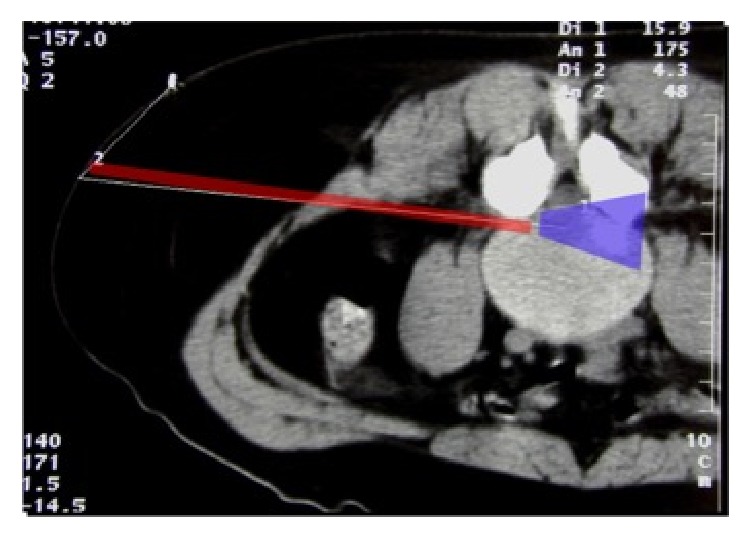
Approach corridor and visual field for transforaminal approach.

This lateral extraforaminal approach enabled the removal of far lateral disc herniations as well as more medially located pathologies because the approach corridor was more parallel to the posterior rim of the annulus (Figure 11).

### 2.4. Lessons Learnt

The indication spectrum for posterolateral and transforaminal endoscopic techniques was limited, which was one of the reasons why endoscopic discectomy remained at a low level of acceptance among spine surgeons in the 1980s and 1990s.

There were other reasons: the variety of instruments was limited, the optical systems were not as good as nowadays, and the technical advantages as compared to microsurgery were small.

### 2.5. Part 3: From a Nondisruptive to a Disruptive Surgical Technology

But what was the missing link or major step? The answer is simple: endoscopy was used in a “dry” environment because the technical advantages of joint arthroscopy were not applied.

Whereas in joint arthroscopy surgical dissection was performed “under water” with continuous irrigation and suction, this principle was not applied in the spine because of the erroneous assumption that irrigation might not be of help or necessary in non-preformed anatomic spaces. The advantages of continuous irrigation (hemostasis, flushing of small bleeding, identification of the bleeding source, better identification of microanatomy, and separation of tissue layers by simple irrigation) were not realized.

Moreover, the technique focussed on lateral extraforaminal approaches, and the most traditional interlaminar approach was believed not to be feasible with such a technique.

This is why “the first wave” of lumbar endoscopic techniques remained a nondisruptive technology.

**Figure 12:**
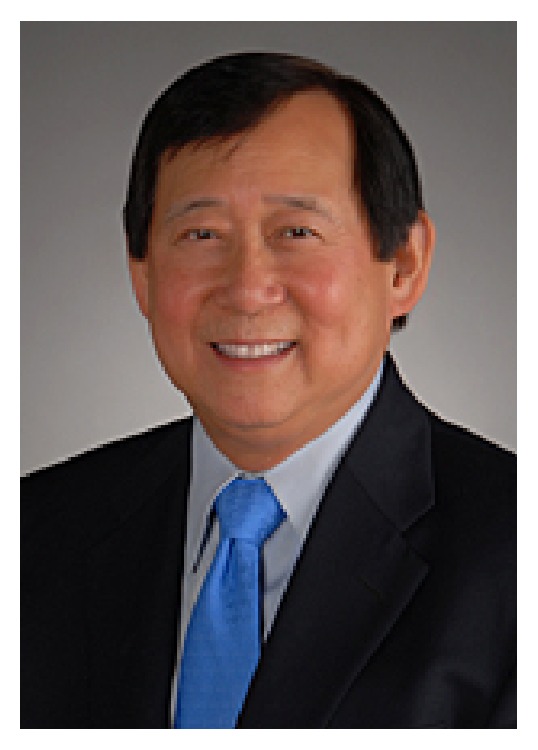
A Yeung: first application of transforaminal approach under continuous irrigation.

Things changed in the late 90s. It was the merit of Anthony Yeung who started to consequently apply arthroscopic technology for transforaminal as well as interlaminar approaches [37, 50, 51] (Figure 12).

There were three major steps, which transferred spinal endoscopy into a disruptive technology:

1. “under-water-dissection”: continuous irrigation reduced intra- and postop bleeding and infection rates and significantly improved visibility of anatomic structures;
2. the range of approaches increased from pure transforaminal or posterolateral to interlaminar because
3. rongeurs, high-speed drills, and other instruments could be used.

Success rates increased and recurrence rates decreased. Rapidly this technology was adopted mainly in Asian countries.

**Figure 13:**
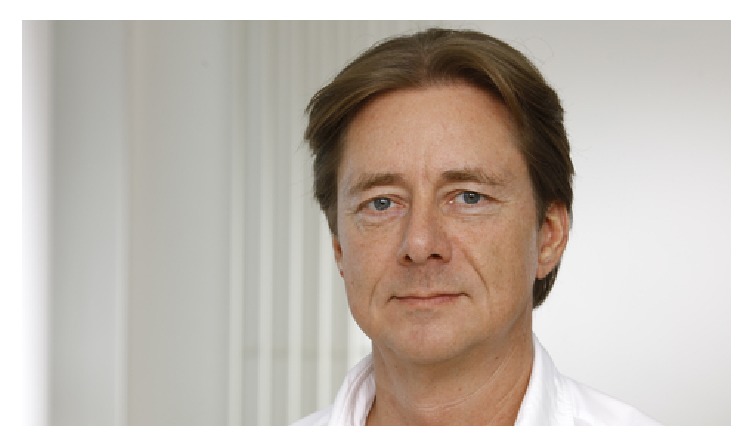
S Rütten: first interlaminar approach and application of arthroscopic technique.

At the beginning of the 2000s it was Sebastian Rütten, a German spine surgeon, who adopted this technology and applied it for interlaminar endoscopic approaches. This significantly enlarged the indication spectrum of this technology (Figure 13).

The current indication spectrum for thoracic and lumbar applications is wide and covers all types of degenerative (and other) pathologies which have been a domain of microsurgical techniques in the past (Table 1)

## 3. Summary

The first attempts of endoscopic lumbar spine surgery date back to the early 1980s. However, only in the last decade this technology has become a disruptive technology with the potential to replace microsurgical techniques especially for degenerative lumbar spine disorders.

The strong input and high acceptance among Asian spine surgeons have triggered a very dynamic clinical and scientific workflow on this topic. A PubMed search for scientific publications on endoscopic lumbar spine surgery shows that more than 80% of the publications have their origin in Asian countries. It has been shown that even though there is a certain learning curve for endoscopic techniques, once the surgeon is familiar with it, he can achieve comparable and sometimes better clinical results as conventional microsurgical operations [52–54].

The complication rates of experienced and well-trained surgeons are low [55].

The iatrogenic collateral damage of the different approaches to the lumbar spine is diminished and most of the procedures can be performed in an outpatient setting [56].

### 3.1. The Future

Today we are in a stage which I would call “microendoscopic blending” where the dynamics of technical improvement of endoscopic techniques suggests that the overlap of indications for this technology vs. microsurgery will step by step convert into a scenario where endoscopic techniques replace microsurgical techniques. The great challenge is the learning curve and the training of young surgeons. The acceptance of this technology is high among young surgeons but it is the task and duty of the protagonists of the older generation, the hospitals, and the scientific societies to develop learning- and training-concepts to shorten learning curves and to improve technical quality and clinical outcomes.

## Figures and Tables

**Table 1:** Indications for full-endoscopic posterior/lateral thoracic and lumbar spine surgery.

(i) Decompression of central and foraminal spinal stenosis [35, 36]
(ii) Decompression of lateral recess stenosis [37]
(iii) Removal of all types of disc herniations incl. difficult cases and recurrent disc herniations [38]
(a) Medial disc herniations [39, 40]
(b) Down migrated disc herniations [41]
(c) Bilateral disc herniations [42]
(d) Recurrent disc herniations [43]
(e) Calcified disc herniations [44]
(iv) Removal of synovial cysts [45]
(v) Removal of epidural hematoma [46]
(vi) Removal of thoracic disc herniations and decompression of thoracic stenosis [47, 48]
(vii) Palliative decompression metastases [49]
